# *MAML2* rearrangement as a useful diagnostic marker discriminating between Warthin tumour and Warthin-like mucoepidermoid carcinoma

**DOI:** 10.1007/s00428-020-02798-5

**Published:** 2020-03-28

**Authors:** Michał Bieńkowski, Michał Kunc, Mariola Iliszko, Alina Kuźniacka, Michał Studniarek, Wojciech Biernat

**Affiliations:** 1 Department of Pathomorphology, Faculty of Medicine, Medical University of Gdańsk, Mariana Smoluchowskiego 17, Gdańsk, 80-214 Poland; 2 Department of Biology and Medical Genetics, Faculty of Medicine, Medical University of Gdańsk, Gdańsk, Poland; 3 Department of Radiology, Faculty of Medicine, Medical University of Gdańsk, Gdańsk, Poland

**Keywords:** Warthin tumour, Warthin-like mucoepidermoid carcinoma, T(11;19) translocation, *MAML2* rearrangement

## Abstract

Warthin tumour is the second most common benign neoplasm of salivary glands. Despite its relatively characteristic histology, it may sometimes mimic other lesions. Here, we report two female non-smoker patients diagnosed with low-grade mucoepidermoid carcinoma with oncocytic epithelium and prominent lymphoid (Warthin-like) stroma and with molecularly confirmed *MAML2* rearrangement. In addition, we screened a consecutive series of 114 Warthin tumour cases by means of *MAML2* break apart fluorescence in situ hybridization to assess its value in differential diagnosis. *MAML2* rearrangement was detected in both mucoepidermoid carcinoma cases, while all Warthin tumours were negative. Taking into account the literature data, Warthin-like mucoepidermoid carcinomas are more frequently observed in women, while a slight male predominance and smoking history are typical for Warthin tumour. In addition, the patients with Warthin-like mucoepidermoid carcinoma were significantly younger than those with Warthin tumour. To conclude, Warthin-like mucoepidermoid carcinoma may usually be suspected based on histology, while the diagnosis can be confirmed by means of molecular assays such as FISH. The investigation of *MAML2* status is particularly advised when Warthin tumour is considered in a young, non-smoking, female patient.

**Electronic supplementary material:**

The online version of this article (10.1007/s00428-020-02798-5) contains supplementary material, which is available to authorized users.

## Introduction

Warthin tumour (WT) is the second most common benign neoplasm of salivary glands. It usually arises as a slow-growing, painless mass in the parotid of male smokers [1]. The mechanism of WT development has not been clearly defined; however, smoking-induced oncocytic metaplasia and salivary gland heterotopia in peri- and intraparotideal lymph nodes are most probably involved [2]. Conventional WT is characterized by a dense lymphoid stroma and cystic spaces lined by bilayer oncocytic epithelium forming papillary projections. Its metaplastic or infarcted variants display coagulative necrosis, fibrosis, and inflammation along with squamous or mucinous metaplasia, but with no cytological atypia or invasive growth pattern [3]; an association with prior biopsy of the tumour has been postulated [4]. In contrast, WT-like mucoepidermoid carcinoma exhibits atypical bilayer oncocytic epithelium with no “typical” bilayer epithelium of WT along with the presence of dense lymphoid stroma and squamoid or goblet-like cells. On the other hand, oncocytic MEC shows the proliferation of oncocytic cells embedded in a desmoplastic stroma infiltrated by a variable number of lymphocytes [5]. Finally, otherwise conventional mucoepidermoid carcinomas frequently show prominent lymphoid proliferation along the infiltrative tumour edge and are sometimes referred to as MUC with tumour-associated lymphoid proliferation [6]. Nonetheless, the microscopic features of the above-mentioned entities overlap to some extent, thus complicating the differential diagnosis, which should include oncocytic papillary cystadenoma, lymphoepithelial lesions (e.g. simple benign lymphoepithelial cyst) and cystic lymph nodes metastases (e.g. so-called Warthin-like papillary thyroid carcinoma) [7] as well as low-grade squamous or mucoepidermoid carcinoma (MEC) [4].

Originally, García et al. reported a series of 12 oncocytic MECs in 2011 [8]. Among these, 5 were described as having Warthin-like histology (all such cases reported to date are summarized in Table 1 [8–14]) and harboured a mastermind-like transcriptional coactivator 2 (*MAML2*) rearrangement. This alteration occurs in > 50% of MECs and correlates with low-/intermediate-grade histology and a better prognosis [15]. Its most common underlying mechanism is t(11;19) translocation, producing CREB-regulated transcription coactivator 1 (*CRTC1*)-*MAML2* gene fusion. Subsequently, Ishibashi et al. coined a new term: Warthin-like MEC (WL-MEC) for a subset of tumours characterized by prominent lymphoid stroma and *MAML2* rearrangement [9]. The distinction between true WT and Warthin-like MEC is crucial as it carries vital clinical consequences. Histopathological features are usually sufficient to obtain the definitive diagnosis; however, recently, Akaev et al. reported a case of MEC with tumour-associated lymphoid proliferation indistinguishable from benign WT by histology and immunohistochemistry [13]. To date, all reported cases of Warthin-like MEC have been associated with *MAML2* rearrangements; thus, the molecular test may provide the definitive answer [12]. On the other hand, it is still unclear whether “classic” WT may exceptionally harbour such translocations.

**Table 1:** Summary of all reported Warthin-like mucoepidermoid carcinoma cases, confirmed by *MALM2* break apart FISH

No.	Sex	Age [years]	Tumour site	Tumour size [mm]	MALM2 break apart FISH	Reference
1	F	68	Parotid	30	Positive	Garcia 2011 [8]
2	F	85	Parotid	NI	Positive	
3	M	50	Parotid	29	Positive	
4	F	46	Parotid	15	Positive	
5	F	64	Parotid	20	Positive	
6	F	28	Parotid	20	Positive	Ishibashi 2015 [9]
7	F	28	Parotid	25	Positive	
8	F	33	Parotid	14	Positive	
9	F	46	Parotid	40	Positive	
10	F	60	Parotid	40	Positive	
11	F	53	Parotid	25	Positive	Hang 2017 [10]
12	F	17	Parotid	NI	Positive	Heatley 2017 [11]
13	M	42	Parotid	31	Positive	Bishop 2018 [12]
14	F	33	Parotid	32	Positive	
15	F	53	Parotid	33	Positive	
16	M	51	Parotid	NI	Positive	
17	F	51	Parotid	12	Positive	
18	F	53	Parotid	25	Positive	
19	F	53	Parotid	12	Positive	Akaev 2018 [13]
20	M	36	Parotid	16	Positive	Zhang 2019 [14]
21	F	31	Parotid	9	Positive	This study
22	F	50	Parotid	16	Positive	

NI – no information

Here, we describe two new cases of low-grade MEC with prominent lymphoid (Warthin-like) stroma and with molecularly confirmed *MAML2* rearrangement. In addition, we screened a consecutive series of 114 WT cases by means of *MAML2* break apart fluorescence in situ hybridization (FISH).

## Case reports

### Case I

**Fig. 1:**
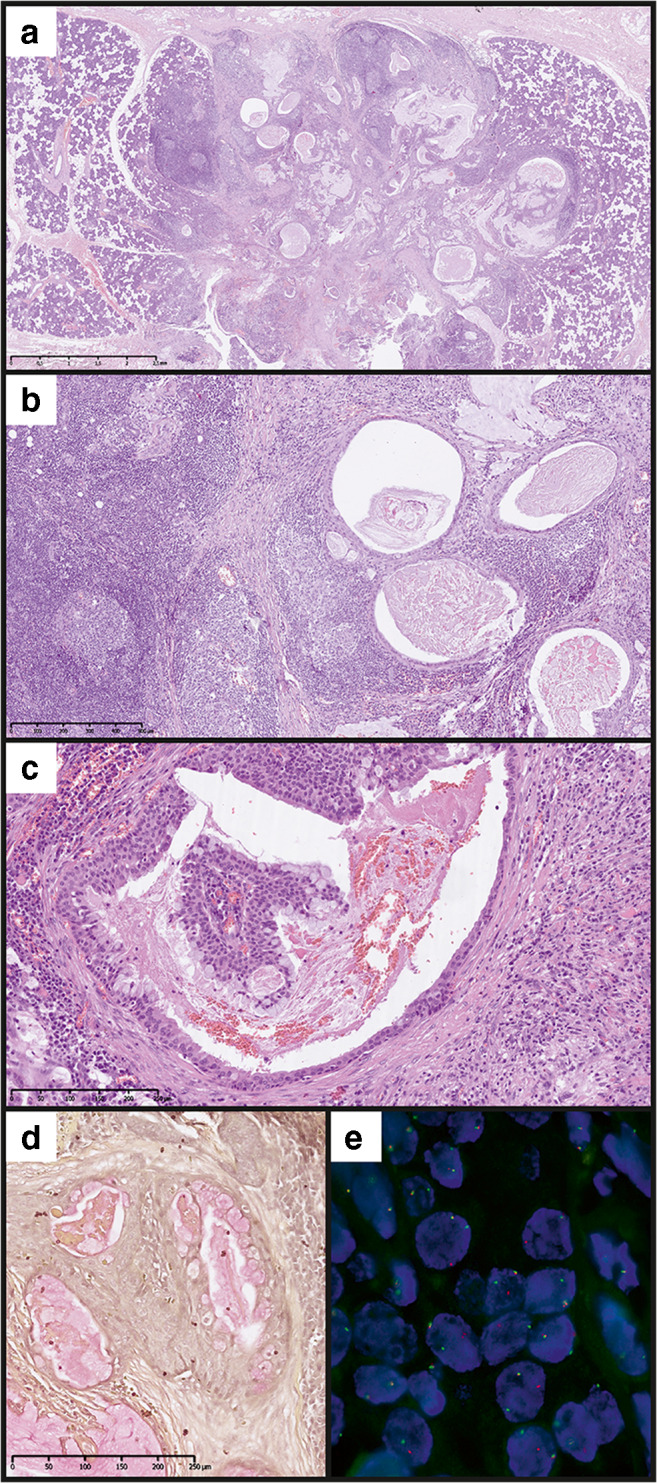
Histology and FISH results of case I. HE images at low (**a**, 2x) and moderate (**b**, 10x; **c**, 20x) magnification, mucicarmine stain (**d**, 20x) and *MAML2* break apart FISH image (**e**, 100x magnification)

A 30-old female non-smoking patient was admitted due to a tumour of the right parotid gland. The lesion was first noted 6 months earlier and caused no discomfort. Of note, 11 years earlier, the patient was diagnosed with Hodgkin’s lymphoma and successfully treated with chemotherapy (no radiotherapy on the neck has been applied). On palpation, the lesion was about 1 cm large, non-movable and not tender; the overlying skin was normal. Grossly, the resected lesion was grey-tan and poorly demarcated from the normal gland. The histological image presented a nonencapsulated tumour with lymphoepithelial growth pattern. It formed numerous cystic structures with variable size and shape, filled with proteinaceous material (Fig. 1a) and surrounded by dense lymphocytic infiltrate with lymphoid follicle formation (Fig. 1b). The epithelium is multilayered and partially oncocytic, containing single scattered mucus-producing cells, confirmed with mucicarmine stain (Fig. 1c, d). No signs of perineurial invasion, necrosis or anaplasia were noted. Thus, mucoepidermoid carcinoma was diagnosed and subsequently assigned as low-grade according to all common grading systems (Modified Healey, AFIP, Brandwein, and Katabi) [16]. *MAML2* rearrangement was confirmed with FISH (Fig. 1f). Due to the resection margins tangent to the tumour tissue, viscerocranial magnetic resonance imaging (MRI) and ultrasound of salivary glands with FNA biopsy were performed, but showed no signs of the residual tumour.

### Case II

**Fig. 2:**
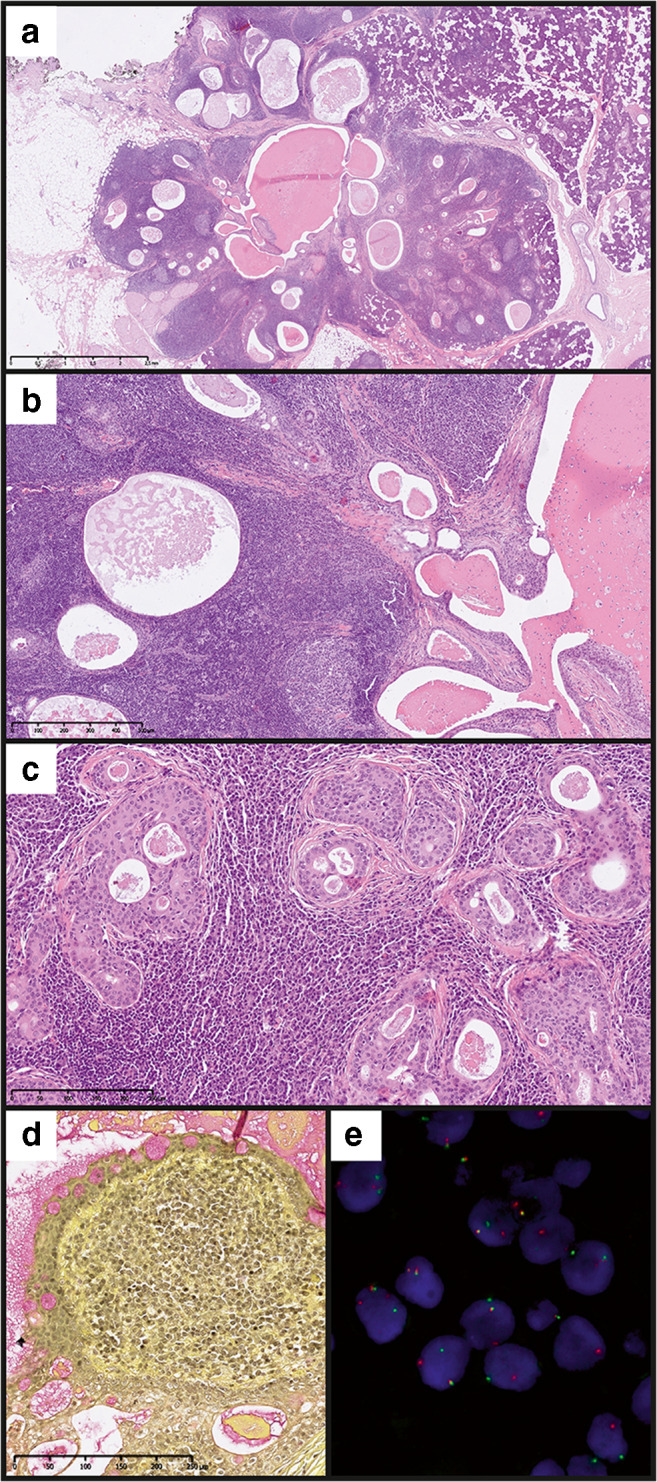
Histology and FISH results of case IIHE images at low (**a**, 2x) and moderate (**b**, 10x; **c**, 20x) magnification, mucicarmine stain (**d**, 20x) and *MAML2* break apart FISH image (**e**, 100x magnification)

A 51-year-old female patient was admitted due to a tumour of the right parotid gland. The lesion was first noted by the patient 7 years earlier and caused no discomfort except for an intermittent otalgia. Fine needle aspiration biopsy was non-diagnostic, while MRI did not allow for differentiation between salivary gland cancers and Warthin tumour (Fig. S1). On palpation, the lesion was about 2 cm large, movable and not tender; the overlaying skin was normal. On gross examination, the lesion was grey-tan and poorly demarcated from the normal gland. The overall histological appearance was similar to the other case. The tumour was nonencapsulated and showed organoid architecture with cystic structures filled with proteinaceous material (Fig. 2a). The accompanying prominent lymphocytic infiltrate forms numerous lymphoid follicles (Fig. 2b). The cysts are lined by eosinophilic squamoid epithelium, and some are subtotally filled with cells showing squamous differentiation mixed with scattered mucus-producing cells (Fig. 2c, d). Thus, MEC was diagnosed, and as in the first case, it was scored low-grade according to the 4 grading systems [16]. *MAML2* rearrangement was confirmed with FISH (Fig. 2f). Due to the incomplete resection, reoperation was performed, and the extended resection margins were free from tumour tissue.

## Materials and methods

### Study group

In addition to the two reported cases of low-grade Warthin-like mucoepidermoid carcinoma, the study group consisted of a consecutive series of 114 Warthin tumour cases diagnosed at the Department of Pathomorphology, Medical University of Gdańsk between 2014 and 2017. Among these, there were 60 males (44 smokers and 4 non-smokers; 91.7%; no data for 12 patients) and 54 females (42 smokers and 6 non-smokers; 87.5%; no data for 6 patients). The median tumour size was 27 mm (range, 6–83 mm). The study was approved by the Bioethical Committee of Medical University of Gdańsk (approval No. NKBBN/207/2019).

### Tissue microarrays

Tissue microarrays (TMA) from WT samples consisting of 2 representative core sections (1 mm in diameter) were prepared using the Manual Tissue Arrayer MTA-1 (Beecher Instruments, Inc., USA). Non-neoplastic tissues served as a negative control and location markers.

### Fluorescent in situ hybridisation

For the purpose of FISH, 4-μm-thick sections were cut from a representative blocks of both MEC cases and from TMAs. Fluorescent in situ hybridisation was performed in parallel to routine diagnostics using ZytoLight SPEC *MAML2* Dual Color Break Apart Probe and ZytoLight FISH-Tissue Implementation Kit (ZytoVision, Bremerhaven, Germany) according to the manufacturer’s protocol. The slides were evaluated using a fluorescent microscope; all epithelial cells within each core were investigated for the presence of the break apart signal.

### Literature search

The search for articles within the PubMed database was performed using the “Warthin-like mucoepidermoid carcinoma” and “Warthin-like MEC” queries (performed on 6 February 2019); subsequently, the reference lists of the included studies were also searched for further articles. Only newly reported cases of MEC with Warthin-like morphology were included in the analysis; thus, 7 studies reporting 20 cases were included (Table 1 [8–14]). Similarly, the PubMed database was searched using “Warthin *MAML2*” and “Warthin t(11;19)” queries (performed on 6 February 2019); subsequently, the reference lists of the included studies were also searched for further articles. Thus, 16 studies reporting the *MAML2* gene status in a total of 162 cases were identified (Table 2 [17–31]).

**Table 2:** Summary of studies investigating the *MALM2* rearrangement in Warthin tumours

Group size	Tumour type	t(11;19) cytogenetic	*MALM2* break apart FISH	*CRTC1-MAML2* RT-PCR	Reference
1	Warthin tumour, NOS	1/1	–	–	Bullerdiek 1988 [17]
1	Warthin tumour, NOS	1/1	–	–	Mark 1989 [18]
9	Warthin tumour, NOS	0/9	–	–	Mark 1990 [19]
13	Warthin tumour, NOS	0/13	–	–	Nordkvist 1994 [20]
13	Warthin tumour, NOS	1/12^a^	–	–	Martins 1997 [21]
7	Warthin tumour, NOS	0/7	0/7	0/7	Martins 2004 [22]
2	Warthin tumour, NOS	1/2	1/2	1/2	Enlund 2004 [23]
					Winnes 2006 [24]
26	Warthin tumour, NOS	–	–	0/26	Okabe 2006 [25]
11	Warthin tumour, NOS	–	–	4/11	Tirado 2007 [26]
2	Warthin tumour, metaplastic*	–	–	2/2*	Fehr 2008 [27]
46	Warthin tumour, NOS	–	–	0/46	
24	Warthin tumour, NOS	–	0/24	0/24	Seethala 2010 [28]
8	Warthin tumour, metaplastic	–	2/8^b^	–	Rotellini 2012 [29]
39	Warthin tumour, NOS	–	0/39	–	Clauditz 2012 [30]
16	Warthin tumour, metaplastic	–	0/16	0/16	Skálová 2013 [31]
4	Warthin tumour, metaplastic	–	0/4	–	This study
111	Warthin tumour, NOS	–	0/111	–	

*NOS* not otherwise specified*reclassified as highly suspicious for mucoepidermoid carcinoma on review^a^diagnosis changed in 2004 to WT ex MEC^b^only in squamous metaplasia

### Statistical analysis

Statistical analysis was performed using R version 3.5.1. with ggplot2 and gridExtra packages for visualisation [32–34]. Continuous variables (age and tumour size) were compared between two groups using Mann-Whitney-Wilcoxon test. Comparison of gender distribution between tumours was performed using chi-squared test.

## Results

**Fig. 3:**
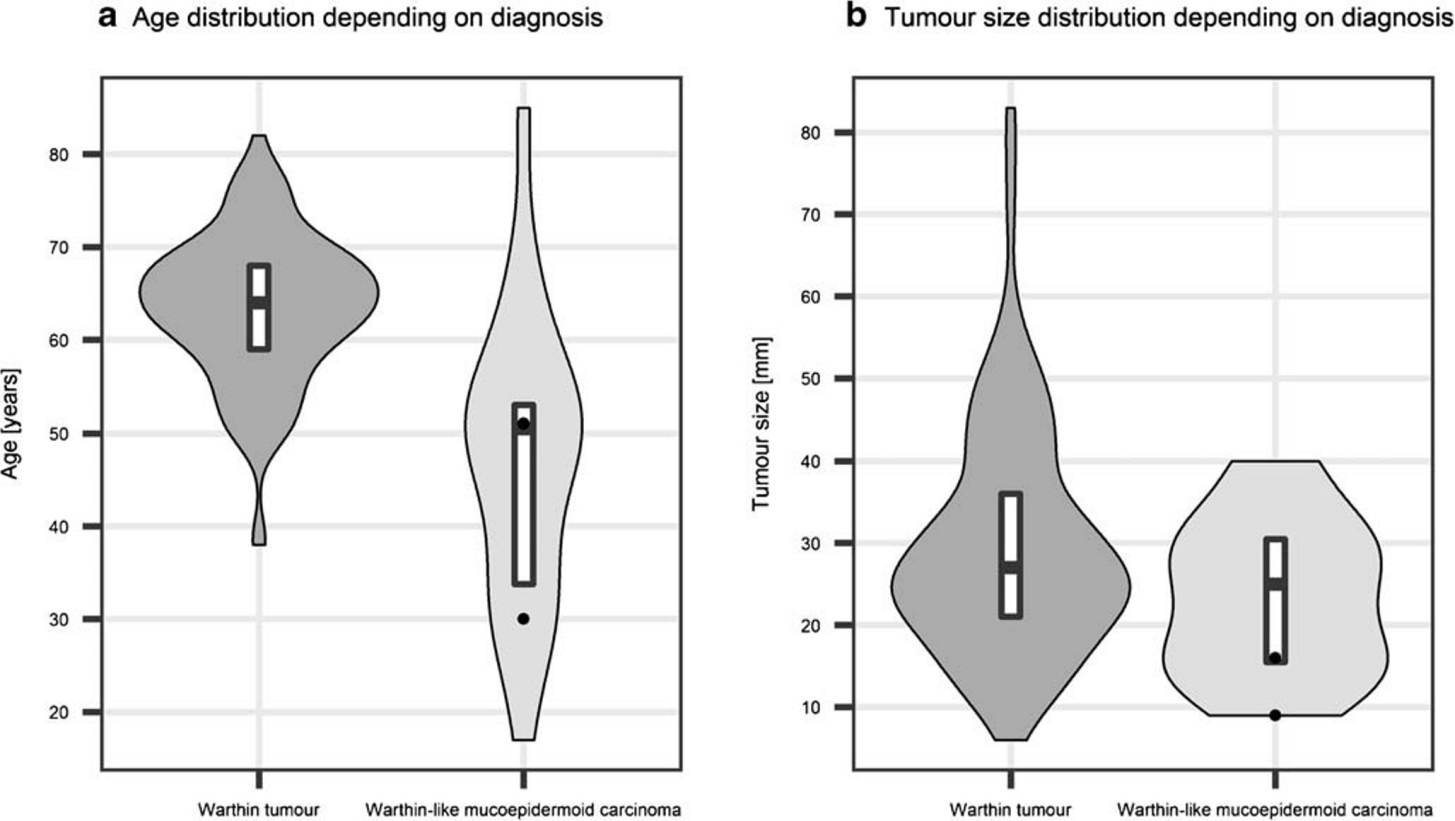
Violin plots presenting age (**a**) and tumour size (**b**) distribution in our series of Warthin tumours (*n* = 114) and all reported Warthin-like mucoepidermoid carcinoma cases (*n* = 22)

*MAML2* rearrangement was detected in both MEC cases (Figs. 1d, 3d). In contrast, all WT cases were negative for the alteration (0/114).

Warthin-like MEC were more frequently observed in women (18/22 reported cases), while a slight male predominance is indicated for WT (*p* = 0.003 when compared to our group of WT; *p* < 0.001 when compared to group reported by Eveson et al. [1, 35]). Additionally, the patients with Warthin-like MEC were significantly younger than those with WT (*p* < 0.001; Fig. 3a). In contrast, there was no difference in the observed tumour sizes depending on the diagnosis (*p* > 0.05, Fig. 3b).

## Discussion

From the clinical perspective, there is a crucial difference between WT and MEC. While the former is entirely benign, the latter may potentially be lethal. Current guidelines indicate the resection for all parotid gland tumours; for Warthin tumours, partial or superficial resection is preferred whenever possible; however, wait-and-scan strategy is sometimes postulated [36, 37]. On the other hand, total parotidectomy, often accompanied by some degree of neck dissection, is the treatment of choice for MEC; in cases with positive margins or high-grade histology, adjuvant radiotherapy should also be considered [36, 38]. In this context, the recently recognized Warthin-like MEC may be particularly problematic. Its cytological appearance suggests Warthin tumour [10], while the radiological features have not been defined; therefore, in most cases, it has been resected as a benign lesion. Albeit on the less aggressive side of the MEC spectrum, Warthin-like MECs still require a closer follow-up, and clear resection margins are more vital, which emphasize its need to be distinguished from WT. Intriguingly, tumours operated on as benign lesions and postoperatively, unexpectedly, diagnosed as malignant are reported to typically follow a benign course [39–41].Warthin-like MEC is a rare and only recently defined entity [9], with few cases described in the literature. Its characteristic morphology along with the *MAML2* rearrangement is crucial for the diagnosis. Before it became a commonly recognized diagnosis, parotid tumours with features of both MEC and WT might have been regarded as MEC ex WT or as a collision tumour [42]. The differential diagnosis of Warthin-like MEC includes metaplastic WT, MEC ex WT, and squamous cell carcinoma with prominent lymphoid response. Metaplastic WT is characterized by nonkeratinizing squamoid cells arranged in cords in necrotic areas, usually accompanied by areas of classic WT [43]. Metaplastic cells may be atypical and grow in pseudoinfiltrative pattern suggestive for malignancy. Perplexingly, mucoid metaplasia may occur as well, which may easily lead to misdiagnosis of MEC [3]. However, metaplastic changes in WT are usually focal and admixed with necrosis, haemorrhages and fibrosis associated with prior biopsy [11]. On the other hand, Heatley et al. described a case of Warthin-like MEC recurring after 4 years as obvious MEC [11]. Detailed evaluation of primary slides revealed a 1-mm distinctive area composed of small cysts lined by attenuated epithelial cells and mucous cells. This case emphasizes the importance of careful examination of doubtful WT cases, which should be confirmed by *MAML2* gene rearrangement detection by FISH. In our cases, the microscopic appearance of the tumours was masquerading WT by formation of lymphoid stroma, papillary architecture and slightly oncocytic cells; however, other features were strongly suggestive for MEC. FISH for *MAML2* confirmed the diagnoses.

Since its discovery [44], the *MAML2* rearrangement, typically resulting from t(11;19) translocation, has been generally considered characteristic for low-grade MEC. This concept was recently challenged by Cipriani et al. [16], who critically revised the histological, molecular and clinical features of a series of MECs. They reported that Brandwein grades were the best predictor of recurrence among the available grading systems. In addition, they suggested that high-grade tumours without the *MAML2* rearrangement are probably high-grade non-mucoepidermoid carcinomas. Both cases presented here were classified as low-grade according to all 4 grading systems (Modified Healey, AFIP, Brandwein, Katabi) [16]. What is crucial, both formation of large cysts and predominance of the cystic component (which are typical for WT-MECs) are the features of low-grade tumours in all systems. On the other hand, none of them may be suitable for MEC variants, since no differences in outcomes were noted between low-, intermediate- and high-grade oncocytic MECs [5].

The comparison of our group of WTs with all reported Warthin-like MEC cases indicates that the latter are observed in significantly younger patients. What is more, our WT cohort comprised no patients younger than 38 years, while all below 45 years were heavy smokers. Similar observations were reported before [9]. Therefore, the status of *MAML2* rearrangement should be investigated when a WT is considered in a young, non-smoking, female patient. Apart from the 2 cases presented here, there is only one WL-MEC case report with a known smoking status, and all 3 patients were non-smokers [14]. Of note, smoking is not considered a strong risk factor for classic MECs [45]. On the other hand, tumour sizes were not significantly different between both investigated tumour types. Still, the group of Warthin-like MECs was relatively small, and these results should be interpreted with caution.

What is noteworthy, some groups reported *MAML2* fusions in classic WT, which challenges the value of *MAML2* as a diagnostic biomarker of Warthin-like MEC [26]. Early cytogenetic studies demonstrated the occurrence of t(11;19) in two random cases of WT [17, 18]. Subsequently, Nodkvist et al. postulated that t(11;19) defines one of three cytogenetic subgroups in WT [20], while Tirado et al. detected the translocation by RT-PCR in 4/11 WT cases [26]. On the other hand, most of the recent studies did not report translocations involving *MAML2* in Warthin tumours (Table 2). Consistently, in the current study, we did not observed any *MAML2* rearrangements in the 114 WT cases. This cohort included 4 cases of metaplastic WT, which had histopathological hallmarks of classic WT along with regions of squamous metaplasia. These findings are in line with the study by Ishibashi and colleagues, who showed that *MAML2* rearrangement-positive metaplastic WT completely lack the typical oncocytic bilayered epithelium and should be reclassified as low-grade MECs [9]. Nevertheless, another study detected split signals indicative for *MAML2* rearrangement in squamous epithelium in two cases of WT with squamous metaplasia, without any accompanying abnormalities in the oncocytic epithelium, lymphocytes and mucinous metaplasia [29]. Recently, Yorita et al. described a case of Warthin tumour with a MEC-like component, in which the lack of *MAML2* rearrangement led to the diagnosis of infarcted WT with metaplastic changes [3]. In contrast, mucoepidermoid carcinoma may potentially develop from WT as postulated by Bell et al. [46]. They reported *MAML2* rearrangements in a subset of WT coexisting with MEC and suggested the possible histogenetic link between these two entities [46]. According to their model, *CRTC1-MAML2* fusion in WT leads to the formation of a more aggressive population, which may transform into MEC. Nevertheless, the cases of MEC ex WT can be relatively easily recognized due to occurrence of transitional areas of squamous metaplasia between the regions of classic WT and obvious MEC [46]. Due to the sparsity of data, it is unknown whether distinguishing between Warthin-like MEC and MEC ex WT has any clinical significance.

It has to be emphasized that this study is the largest reported screening of Warthin tumours by means of break apart FISH to detect the *MAML2* rearrangement. The application of TMAs for FISH might be regarded as a limitation; however, others have previously demonstrated the reliability of such an approach as confirmed by real-time polymerase chain reaction (RT-PCR) [28]. Moreover, the classical bilayer architecture of WTs could readily be appreciated by fluorescent microscopy, while all cores from Warthin-like MEC presented the translocation.

To conclude, Warthin-like MEC may usually be suspected based on histology, while the diagnosis can be confirmed by means of molecular assays such as FISH. In contrast, classic and metaplastic WTs containing the characteristic bilayered oncocytic epithelium are typically not associated with *MAML2* rearrangement and usually affect older patients.

## Electronic supplementary material

ESM 1 (PNG 2738 kb)

High resolution image (TIF 4978 kb)
