# Supplementary figures and images for: MAML2 rearrangement as a useful diagnostic marker discriminating between Warthin tumour and Warthin-like mucoepidermoid carcinoma
Source: Virchows Arch. 2020 Mar 28;477(3):393–400. doi: 10.1007/s00428-020-02798-5 (PMC7443186; doi:10.1007/s00428-020-02798-5)

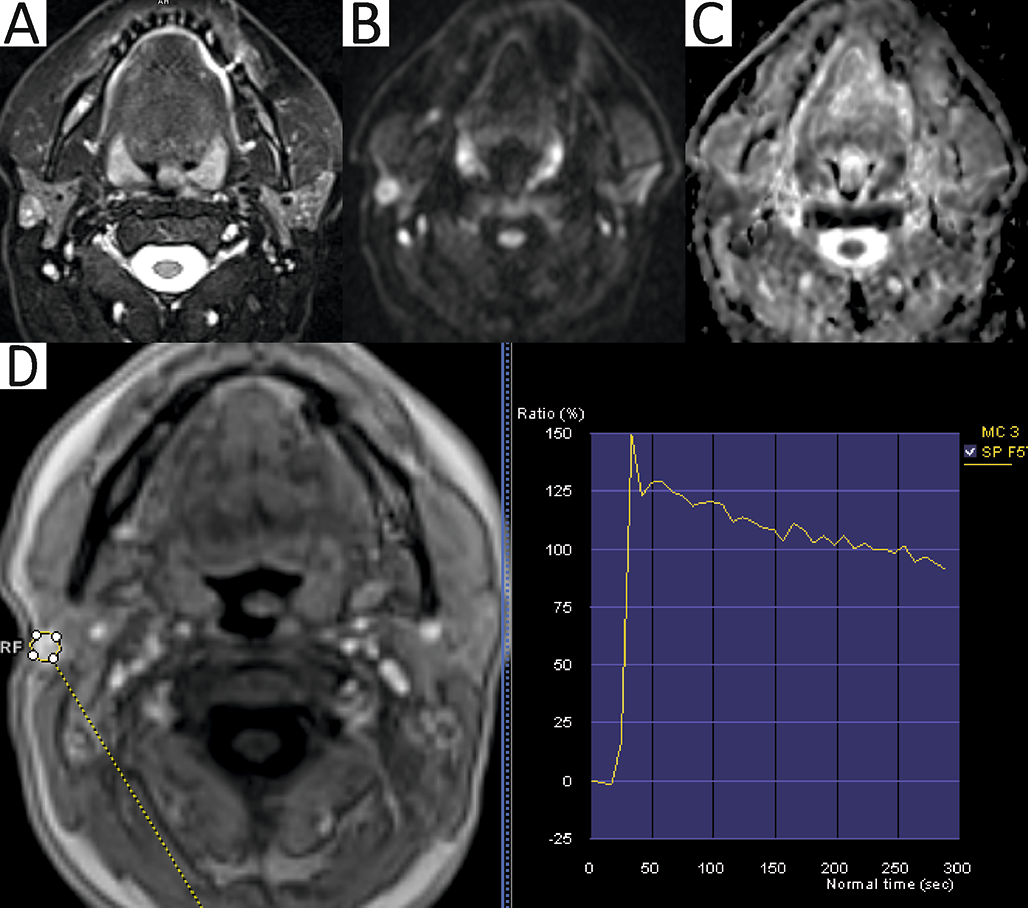

Supplement: Supplementary file 1 — (PNG 2738 kb) [file 428_2020_2798_Fig4_ESM.png]

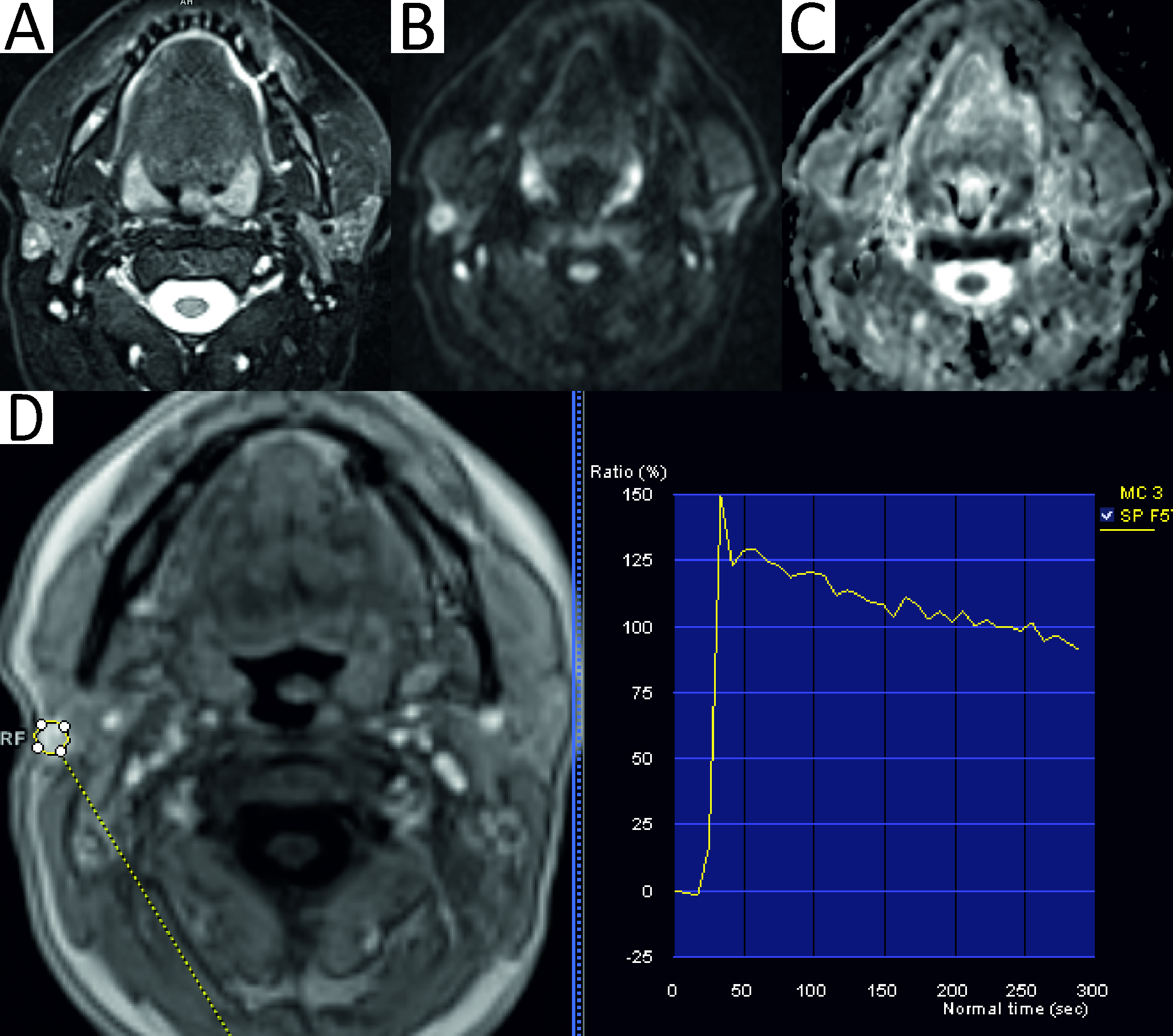

Supplement: Supplementary file 2 — High resolution image (TIF 4978 kb) [file 428_2020_2798_MOESM1_ESM.tif]
